# Community Engagement in Youth Violence Prevention: Crafting Methods to Context

**DOI:** 10.1007/s10935-016-0428-5

**Published:** 2016-03-11

**Authors:** Susan Morrel-Samuels, Martica Bacallao, Shelli Brown, Meredith Bower, Marc Zimmerman

**Affiliations:** School of Public Health, Michigan Youth Violence Prevention Center, University of Michigan, 1415 Washington Heights, Ann Arbor, MI 48109-2029 USA; North Carolina Center for Excellence in Youth Violence Prevention, The University of Kansas, Twente Hall, Lawrence, KS 66045-3129 USA; Colorado Academic Center for Excellence in Youth Violence Prevention, University of Colorado, Boulder, 1440 15th St., Boulder, CO 80302 USA; North Carolina Center for Excellence in Youth Violence Prevention, University of North Carolina, Chapel Hill, 325 Pittsboro St., Chapel Hill, NC 27599 USA

**Keywords:** Youth, Violence, Prevention, Community, Engagement

## Abstract

The purpose of the Youth Violence Prevention Centers (YVPC) Program at the Centers for Disease Control and Prevention is to reduce youth violence in defined high-risk communities through the implementation and evaluation of comprehensive, evidence based prevention strategies. Within this common framework, each YVPC varies in its structure and methods, however all engage communities in multiple ways. We explore aspects of community engagement employed by three centers that operate in very different contexts: a rural county in North Carolina; a suburban area of Denver, Colorado; and an urban setting in Flint, Michigan. While previous research has addressed theories supporting community involvement in youth violence prevention, there has been less attention to the implementation challenges of achieving and sustaining participation. In three case examples, we describe the foci and methods for community engagement in diverse YVPC sites and detail the barriers and facilitating factors that have influenced implementation. Just as intervention programs may need to be adapted in order to meet the needs of specific populations, methods of community engagement must be tailored to the context in which they occur. We discuss case examples of community engagement in areas with varying geographies, histories, and racial and ethnic compositions. Each setting presents distinct challenges and opportunities for conducting collaborative violence prevention initiatives and for adapting engagement methods to diverse communities. Although approaches may vary depending upon local contexts, there are certain principles that appear to be common across cultures and geography: trust, transparency, communication, commitment. We also discuss the importance of flexibility in community engagement efforts.

## Introduction

The purpose of Youth Violence Prevention Centers (YVPC) Program, funded by the Centers for Disease Control and Prevention (CDC), is to reduce youth violence in defined high-risk communities by means of the implementation and evaluation of comprehensive, evidence-based prevention strategies (U.S. Department of Health and Human Services, 2010). Within this common framework, the structure and methods of each YVPC varies; however all engage communities in multiple ways. We explore aspects of community engagement employed by three centers that operate in very different contexts: a rural county in North Carolina, a suburban area of Denver, Colorado, and an urban setting in Flint, Michigan.

The geographic areas served by the three YVPCs described in this article differ in size, population, and racial and ethnic composition. The North Carolina Center for Excellence in Youth Violence Prevention (NC-YVPC) operates in Robeson County, which is largely rural and has a very diverse population of American Indians, African Americans, Latinos and European Americans. The Colorado Academic Center for Excellence in Youth Violence Prevention (CO-YVPC) serves the Montbello neighborhood of Denver. Originally designed as a suburban enclave within the city, this neighborhood is home to large Hispanic and African American populations. The Michigan Youth Violence Prevention Center (MI-YVPC) focuses on an approximately two square-mile area of Flint, MI. The population in the MI-YVPC intervention area is predominantly African American.

Every YVPC is expected to collaborate with community partners and local organizations. Beyond this requirement, however, the extent and form of community engagement activities are within the discretion of the grantees, allowing each site to tailor its approaches to the unique needs and histories of its localities and the focus of its intervention strategies. As a result, each of the Centers described in this article employs an array of methods for community engagement in keeping with its discrete context and goals. These include strategies to engage local organizations, adult residents, and youth. The purpose of this paper is to describe the practical dynamics of implementing community engagement in youth violence prevention efforts and illustrate how they relate to principles outlined in the literature on effective community-academic partnerships.

## Background

The National Institutes of Health Director’s Council of Public Representatives defines community engagement as:

…a process of inclusive participation that supports mutual respect of values, strategies, and actions for authentic partnership of people affiliated with or self-identified by geographic proximity, special interest, or similar situations to address issues affecting the well-being of the community of focus. (Ahmed & Palermo, 2010, pg. 1383)

Youth violence prevention initiatives offer both challenges and opportunities for community engagement. Kim-Ju et al. (2008) note that the complex etiology of youth violence requires an interdisciplinary, cross sector approach to address risk and protective factors at multiple ecological levels. This entails bringing together individuals and organizations that may not typically collaborate (e.g., public health, law enforcement, youth services, neighborhood groups, schools). Participants from these diverse sectors are likely to have different orientations towards youth violence concerning, for example, the importance of prevention versus enforcement or the efficacy of universal as opposed to targeted interventions (Krug, Mercy, Dahlberg, & Zwi, 2002). Additionally, there may be legacies of mistrust among community members and institutions to overcome (Griffith et al., 2008). Identifying and addressing these barriers is important because several theoretical frameworks indicate that community engagement and cohesion are critical components of youth violence prevention strategies.

Social ecological and social disorganization theories posit that individuals are affected by their contexts and that they, in turn, exert influence on those contexts (Aber, Bennett, Conley, & Li, 1997; Sameroff et al., 1987; Sampson, Morenoff, & Gannon-Rowley, 2002). Previous research has documented that particular aspects of social organization (e.g., social support, trust) and presence of community organizations (e.g., neighborhood groups, youth-serving organizations) are inversely associated with youth violence (Gorman-Smith, Henry, & Tolan, 2004; Kawachi, & Kennedy, 1999; Peterson, & Newman, 2000; Sampson et al., 2002; Taylor, Gottredson, & Brower, 1984). Neighborhood contexts are shaped by community norms and values that influence behavior (Sampson et al., 2002). Additionally, while specific violent acts involve individuals, the presence of violence affects entire communities on multiple levels: psychologically, through fear and mistrust (Perkins & Taylor, 1996; Kruger et al., 2007); physically, by restricting freedom of movement (Loukaitou-Sideris & Eck, 2007; Stafford, Chandola, & Marmot, 2007); and economically, by deterring businesses and reducing property values (Hipp, 2011; Oh, 2005). Thus, those affected by community violence have much to gain or lose from youth violence interventions and may contribute to crafting solutions by sharing their first-hand knowledge and experiences.

Over the past two decades there has been increasing interest in the potential of forming community partnerships to address complex public health issues. Several authors have proposed guidelines and principles to facilitate the establishment and maintenance of effective partnerships (Baker, Homan, Schonhoff, & Kreuter, 1999; Buys & Bursnall, 2007; Green, Daniel, & Novick, 2001; Plowfield, Wheeler, & Raymond, 2005). While there are variations in the principles and practices outlined in the literature, several common themes emerge. Multiple authors identify trust, transparency, communication and commitment as critical values underlying successful partnerships (Baker et al., 1999; Green et al., 2001; Plowfield et al., 2005).

Many of the specific recommendations articulated in the literature fall within these four broad categories. Plowfield et al. (2005) emphasize trust-building through investing adequate time in partnership development and operating under the assumption that all parties are working in good faith to address the chosen problem. Trust is enhanced by transparency, which includes being clear about the extent and nature of community involvement, and acknowledging and respecting different agendas among partners (Baker et al., 1999). Other aspects of transparency include agreeing on roles, norms and processes for partnerships using input from all partners and developing common missions, goals and outcomes (Green et al., 2001). Multiple authors address aspects of communication that are critical to maintaining successful partnerships. Plowfield et al. (2005) note the importance of tactful, direct communication when voicing needs and concerns, while Green et al. (2001) emphasize the necessity of attending to feedback from all partners. Clear communication is of particular importance as partnerships mature and transition to new stages (Baker et al., 1999). While trust, transparency and communication are essential to establishing community-academic partnerships, commitment is a key component in achieving their sustainability. Commitment may be expressed through building on identified strengths and assets within a community and by using existing structures, such as schools and worksites, to implement partnership strategies (Green et al., 2001). Identifying talented leaders from the community (Plowfield et al., 2005) and establishing strong relationships with local institutions (Green et al., 2001) are other dimensions of commitment that help lead to sustainability.

Baker et al. (1999) emphasize the importance of identifying best processes and models based upon the nature of the issue and the intended outcome. An example of such a model, developed specifically for violence prevention, is Communities That Care (CTC, Hawkins et al., 2008). CTC is a coordinated series of strategies designed to help community organizations, researchers, and other key stakeholders establish coalitions to address adolescent problem behaviors. The CTC model was tested in a randomized controlled trial that examined effects on risk behaviors among students at 6 and 8 years after implementation (Hawkins et al., 2012; Hawkins, Oesterle, Brown, Abbott, & Catalano, 2014). At 6 years, the increase in targeted risks among youth was less rapid in the intervention communities and the incidence of past year delinquent and violent behavior was lower. At 8 years, students in the intervention communities were less likely to have ever engaged in delinquency or committed a violent act. There were no differences, however, between intervention and control communities in the prevalence of past-year delinquency or violence. The Colorado case example described in this paper applies the CTC model to the Montbello community of Denver.

In the case examples that follow we use observations from three YVPCs to illustrate how community engagement principles of trust, transparency, communication and commitment have been applied. We have categorized each example based upon its closest connection to a principle, even though these concepts are closely related and are sometimes difficult to isolate in practice. We detail the barriers and facilitating factors that influenced implementation, and describe lessons learned from working with violence prevention coalitions in the three settings. Finally, we provide recommendations for applying community engagement principles in violence prevention initiatives. The examples are drawn from the authors’ experiences as participant-observers. Jorgensen (2015) describes participant observation as a method of investigation in which a researcher participates in a particular setting while gathering information. The authors’ reflections are based upon observations of numerous coalition meetings and discussions with YVPC staff and community members at each Center.

## Case Examples

### Rural Context: North Carolina Youth Violence Prevention Center (NC-YVPC)

#### Setting

Established in 2010, the NC-YVPC is a collaboration among faculty from the University of North Carolina (UNC) at Chapel Hill’s School of Social Work, the UNC Jordan Institute for Families, the UNC Injury Prevention Research Center, and community agencies in Robeson County, including the Robeson County Health Department, the juvenile court system, the Department of Social Services, and Public Schools of Robeson County. NC-YVPC serves the youth and residents of Robeson County, NC, one of the most ethnically/racially diverse rural counties in the United States. Additional information about the NC-YVPC may be found in Matjasko, Massetti, and Bacon (2016) and Kingston, Bacallao, Smokowski, Sullivan, and Sutherland (2016).

Robeson County is unique in that it has a large population of American Indian youth (40 % of the County’s total population of 135,000 is Lumbee, 24 % are African Americans, 9 % are Latinos, and 27 % European Americans; U.S. Census, 2010) and has a homicide rate that is five times the national average. Robeson County, spanning 925 square miles, is also among the most socioeconomically disadvantaged counties in North Carolina and the United States, with a child poverty rate of 47.8 % in 2013. Despite these challenges, the Robeson County community has several community assets including the oldest rural health department in the nation and an extensive network of community churches and agencies that support the exceptionally diverse population. The county’s expansive rural setting and rich mixture of cultures present unique challenges and opportunities for community engagement.

#### Community Engagement Processes

##### Trust

Lack of trust is perhaps the largest obstacle for community-based intervention researchers. Trust is an asset that must be earned with each new partnership project, especially in a vulnerable community. In Robeson County, residents felt that previous researchers had come, collected data, published negative findings and left, giving the community a poor experience of feeling like “lab rats.” NC-YVPC had to address this negative legacy by showing dedication, integrity, and reliability. When NC-YVPC received funding in 2010, the partnership with Robeson County was in its infancy. This was the first time these partners from the University of North Carolina and the county had worked together. In this large rural area, the university staff needed to engage a wide range of community representatives within various organizations and service sectors to plan, make decisions, implement and evaluate a package of evidence based programs. In the Center’s initial year of planning and partnership development, trust was one of the first issues to address.

Rather than focusing on one or two partners, the NC-YVPC brought together a Youth Violence Prevention Council with wide community representation to assist in decision-making. Members of this council were invited from major agencies that served youth in different capacities: the juvenile justice system, the public health department, tribal government, mental health agencies, the Division of Social Services, school district administration, the county sheriff’s department, and the Boys & Girls Club. One of the strengths of the rural context was that many of these providers knew each other and were willing to come together to represent the county. The NC-YVPC’s Youth Violence Prevention Council was used to further the partnership processes articulated by Green et al. (2001). Council members worked to develop the mission and goals of the partnership, identified community assets and challenges, and created norms, roles, and communication processes to support their work together. Through these meetings, agency representatives and university partners got to know each other. Community representatives saw that their ideas and suggestions were valued, building trust in the working relationships among the partners.

Community engagement is vital to the success of a comprehensive, community-level youth violence prevention initiative, however, the engagement process in a large rural county is a time consuming and labor intensive effort that requires a high level of involvement, participation, and visibility. Particularly in the initial formative stages of partnership development, it was crucial to understand community norms, history, values, and interpersonal social networks in order to build trust.

##### Transparency

To create and sustain the NC-YVPC coalition, the researchers and staff needed to be inclusive and transparent, and to communicate clearly and consistently. It was critical for partnership development to simultaneously address process and product. While relationship building was in progress during the planning year, partners from the Public Schools of Robeson County worked with university researchers to collect needs assessments from more than 4000 middle school youth (a random sample of 40 % of 6th through 8th grade adolescents) using the School Success Profile-Plus (Smokowski, Cotter, Robertson, & Guo, 2013). Summary profiles for each school across the county were presented to the Youth Violence Prevention Council. Council partners worked together to identify and prioritize risk factors to target with prevention programming. This was a critical shared activity; extensive discussions triangulated the needs assessment data with community members’ lived experiences, resulting in a short list of risk factor targets that everyone agreed upon. After a year of this planning process, the Youth Violence Prevention Council identified high levels of: (1) school violence and bullying; (2) parent child conflict; and (3) juvenile arrests. These risk factors aligned with both community residents’ concerns and researchers’ needs assessment data.

The second shared activity was reviewing a menu of potential evidenced based prevention programs, matching these with community strengths and implementation partners. When planning the content of the comprehensive youth violence prevention initiative, suggestions from both researchers and community members were reviewed. The Council chose Positive Action (Flay, Allred, & Ordway, 2001), a universal prevention program for middle schools designed to address bullying, Parenting Wisely (Cotter, Bacallao, Smokowski, & Robertson, 2013), to address parent–child conflict among high risk families, and Teen Court, to divert adolescent first offenders from the juvenile courts and to re-engage them with community service using a restorative justice framework (Butts & Buck, 2000). Based on community members’ recommendations (rather than the standard archives of evidenced-based programs), Teen Court became a core component of the comprehensive prevention package. It also became the most sustainable part of the package because community members were highly invested in its success.

The resulting multilevel, multi-sector prevention package targeted different ecological systems (youths, peer networks, families, and schools) and utilized specific community partners to engage in implementation (the school system for Positive Action, the network of community agencies for Parenting Wisely, the courts and juvenile justice partners for Teen Court). This ecological approach maximized inclusion in the comprehensive initiative because programs targeted the individual needs of students, parents, school personnel, and juvenile justice system workers. The burdens and benefits of implementation were shared across diverse agencies, creating a tight knit “safety net” for adolescents and their families to participate in the comprehensive initiative from different points of entry.

The shared activities of needs assessment and intervention selection were critical for all partners because trust, transparency, and mutual investment were demonstrated by action as well as discussion. Moving towards the articulated goals by creating a specific plan reinforced the shared belief that the partnership was worthwhile.

##### Communication

University researchers learned to adjust their communication styles to be more consistent with the norms and values in Robeson County. Residents of this rural county trusted insiders over outsiders. There was wariness towards people from outside the county whom they did not know. Relationship-building interactions happened outside of formal meetings and were often the result of interpersonal social ties and networks in the community. Informal communications and flexibility in working with diverse social networks were critical in managing obstacles that arose. In one example from the beginning of the project, a school principal with whom the NC-YVPC staff wanted to collaborate was difficult to reach. His meeting schedule was usually booked. While working with another principal in a nearby town, NC-YVPC staff found that he was friends with the principal with whom they had been trying to connect. The collaborating principal suggested that the next time he met his friend for lunch, the NC-YVPC staff members stop by and he would introduce them. He told his colleague about the positive experiences he had working with the initiative. This communication from a trustworthy source helped to build a relationship that had been challenging to initiate.

In another example, university partners quickly learned that when making presentations throughout the county, it was ideal to include a community member (i.e., an insider) who would deliver part of the presentation. The community representative became an ambassador for the initiative. Recognizing the importance of local relationships also provided the impetus for hiring staff members who lived in the county. It was important to be cognizant of these communication dynamics while being a consistent presence in the community.

##### Commitment

It was critical to the Robeson County community members that the partnership would seek long-term change. Even with community involvement in initial program selection and implementation it was important to continue to attend to cultural contexts and community preferences, and to remain flexible to meet the diverse needs of program participants. Continued attention to intervention implementation was a fundamental aspect of the researchers’ commitment to meeting the needs of the community. NC-YVPC staff and implementation partners used the insights gained from community relationships and local understanding of the county to tailor aspects of the intervention initiative. To culturally adapt the programs to this community context, staff included examples in the Positive Action middle school curriculum that were relevant to the diverse, rural context. References to Lumbee culture were inserted in discussions of the Positive Action curriculum’s content. In the parenting program, video content was supplemented with discussions of group member’s experiences. Facilitators helped parents role play difficult situations that they had experienced with their teenagers to practice parenting skills from the curriculum. These adaptations were important to show respect for community norms, demonstrate flexibility, and show commitment to maintaining positive relationships with community partners and participants. Thus, cultural adaptations not only boosted “fit” with the target community, but also fostered and strengthened community respect, rapport, and engagement. These adaptations demonstrated flexibility, showed a commitment to local values, and maximized potential impact because specific community issues and concerns were included in the intervention content.

By working together in selecting, implementing, adapting and evaluating youth violence prevention programs over 5 years, this experience of university-community collaboration refashioned many of the negative views of research that community partners had from the past.

### Suburban Context: Colorado Academic Center for Excellence in Youth Violence Prevention (CO-YVPC)

#### Setting

The CO-YVPC is based in the Center for the Study and Prevention of Violence (CSPV) at the University of Colorado Boulder. Guided by its founding director, Delbert Elliot, CSPV has provided assistance to groups committed to understanding and preventing violence, particularly adolescent violence. The CO-YVPC’s purpose is to promote positive youth development and reduce youth violence in the Denver neighborhood of Montbello through a coordinated, community-wide effort. Additional information about the CO-YVPC may be found in Matjasko et al. (2016) and Kingston et al. (2016).

Montbello is a neighborhood approximately 4.5 square miles in size and situated in the far northeast suburban area of Denver, Colorado. According to the 2010 Census, its population of approximately 31,000 is largely Hispanic and African American. Over time, the community’s race and ethnicity demographics have changed from mostly African American residents at its inception, to a majority of Hispanic residents. Montbello has always been steeped in a rich cultural identity, and in recent years it has been challenged by a fluctuating socioeconomic climate. A report from the Piton Foundation (2011) found that over 90 % of Montbello’s youth participated in the free and reduced lunch program at school (a socioeconomic indicator). Further, the area is served by only a small (though growing) number of non-profit organizations. This setting, coupled with relatively high levels of youth crime and other problem behaviors, suggested that Montbello would be ideal for the support the CO-YVPC could bring.

#### Community Engagement Processes

##### Trust

Existing local and city champions from Montbello’s Families Forward Resource Center, Denver’s Public Safety Youth Programs Office, the Crime Prevention and Control Commission, and area churches supported reducing levels of youth violence and promoting positive youth development. This early support became the foundation for the work of the CO-YVPC. Some community members, however, expressed a lack of trust in researchers coming into their community. The phrase “hit and run research” was coined early on and led to a sharper understanding about the importance of consulting participants in every decision to be made. Prior to the start of the CO-YVPC, Denver’s far northeast neighborhoods, Montbello included, underwent a significant school turnaround effort to address some of the educational deficits the area faced. There continued to be lingering mixed feelings about the success of the efforts, and some residents felt as though they were not listened to with respect about how to improve failing schools. To overcome this legacy of mistrust, local community engagement and ongoing collaboration were woven into the processes by which the work of the Center was completed. The CO-YVPC chose to use the Communities That Care (CTC) operating system (Hawkins et al., 2008) in its work within the Montbello community to meet its goal of local community engagement and collaboration. The CTC strategic planning system was chosen for its evidence-based structure and obligation to include significant community involvement. The CTC model created an atmosphere that blended the work of prevention science and community organizing. It is grounded in research that advocates using a public health approach, requires a comprehensive, community-wide strategy focusing on demonstrated predictors of problem behaviors and positive youth outcomes, and uses tested, effective programs, and policies (Hawkins & Catalano, 2005). The CTC system provided a structure for building trust within the context of the CO-YVPC community partnerships. Community members and partners were significantly engaged in the decision-making process and final approval of all decisions came from the members of the Community Board, as individuals with the most to gain or lose from potential interventions.

##### Transparency

The CO-YVPC employed the CTC five-phase system that was transparent and engaged the community at every step. It involved assessing the community for readiness, creating Key Leader and Community Boards, collecting a comprehensive body of data from youth and parents, making data driven decisions, creating a Community Action Plan, and implementing evidenced-based programs (Hawkins & Catalano, 2005). Transparency was demonstrated by engaging partners (both residents and city officials) in analyzing collected data and identifying risk and protective factors, as well as choosing the programs to be funded, all key elements of the CTC model. The Community Board ensured that the programs were integrated successfully into existing service agencies and established a system to monitor program implementation and assess expected outcomes. Creating the Community and Key Leader Advisory Boards and maintaining transparency within them has helped to keep the community aware and engaged in the process.

##### Communication

Initially, the CO-YVPC experienced limited engagement from the Hispanic community in Montbello, a challenge that was difficult to overcome. Other initiatives, efforts, and agencies within the community had previously worked to address this challenge with varying degrees of success. Approximately 59 % of Montbello’s population is Hispanic (Piton, 2011). Thus, engaging the Hispanic population was a necessary prerequisite to effectively impacting the population this initiative was intended to serve. The CO-YVPC began its attempts at increasing Hispanic engagement with regular communication efforts that included participating in community events and inviting interested parties to join in planning and related activities. For example, a local church with a large Hispanic congregation invited our initiative to participate in their biannual event at which community support organizations presented agency information and provided relevant resources to their congregation. The CO-YVPC attended and used this communication opportunity to recruit paid interviewers, as well as to provide information about violence prevention. Additionally, a local high school hosted the first Latino Education Summit in the Montbello neighborhood and invited CO-YVPC to participate by staffing a resource table. Most recently, CO-YVPC has made strides in Hispanic engagement due, in part, to partnering with the aforementioned local church with a primarily Spanish-speaking congregation to host one of the evidenced-based programs initiated through the Community Action Plan. The CO-YVPC continues to build relationships with the Hispanic community through proactive communication as program implementation is woven into the community fabric and the positive impact of the work becomes clearer to Montbello residents.

##### Commitment

Examples of how the CO-YVPC has demonstrated commitment to the community are numerous. From the creation of the vision statement that drives this work, *“A self*-*empowered community that we are proud of,”* to the recruitment and hiring of specific positions within the initiative, CO-YVPC has been successful and intentional in engaging partners at the community, city, and state levels. The Community Site Manager, data collectors, and facilitators for at least one of the evidenced-based programs were hired from the community to work within the community. Employing residents not only built capacity and showed commitment, it also created an environment in which sustainability could thrive.

Montbello community members made it clear that they did not want to feel used by “research,” but instead wanted this initiative to become rooted in the community with positive impacts. As part of the CTC operating system, there is a committee embedded within the Community Board dedicated specifically to sustainability. The CO-YVPC’s Sustainability Committee’s function lies in working with supporters to understand which aspects of CO-YVPC should and can be sustained. Part of their thoughtful planning has included a better understanding of what a prevention infrastructure should look like and how that fits into Montbello’s current and future cultural, financial, social, and agency priorities. The Sustainability Committee members recognized that the short term success experienced has an improved chance of sustainability in the long-term with ongoing commitments from residents, stakeholders, and partners. The Center’s long-term success in Montbello depends upon community engagement and investment at every level of this youth violence prevention initiative.

### Urban Context: Michigan Youth Violence Prevention Center (MI-YVPC)

#### Setting

The MI-YVPC, established in October of 2010, is based at the University of Michigan School of Public Health and includes partner organizations in law enforcement, local public health, health systems, faith-based organizations, youth services, land use, and community groups. From 2010 to 2015, the MI-YVPC operated in a defined neighborhood of the city to support six violence prevention programs at the individual, family and community levels (Youth Empowerment Solutions, Fathers and Sons, Sync, Targeted Outreach Mentoring, Clean and Green, and Community Mobilization). These programs encompassed both primary and secondary prevention approaches. Additional information about the MI-YVPC may be found in Matjasko et al. (2016) and Kingston et al. (2016).

The MI-YVPC programs were offered in an area of Flint that is primarily residential, and includes many vacant and deteriorated properties. During the 1960s, the City of Flint and surrounding Genesee County was one of the most prosperous metropolitan areas in the nation due to many high paying manufacturing jobs at multiple car and truck factories. Since 1970 Genesee County has lost ninety percent of these auto industry jobs. Flint now ranks well below most other Michigan communities on many socioeconomic indicators (U.S. Census, 2014).

Although Flint faces economic and social challenges, the city also benefits from assets that provide a firm foundation for community change. Results of a 2011 community survey (Prevention Research Center of Michigan, 2011) indicated that Flint residents were more likely than others in their county to participate in collective activities such as neighborhood beautification projects, crime watches, or other actions with neighbors to address neighborhood problems. Flint also possesses many non-profit organizations whose missions include supporting youth. The MI-YVPC built upon this tradition of community involvement in its efforts to prevent youth violence.

#### Community Engagement Processes

##### Trust

Universities and other large institutions often face legacies of community mistrust related to perceived and real inequities or previous negative experiences. Flint has been the subject of many research studies and media reports, and residents were sensitive about its image as a place of deterioration and violence. To build trust, the MI-YVPC connected with community organizations early in the planning process to seek their advice and support. This community engagement work began well before the proposal was funded. The investigators had well-established relationships with key organizations in the community through previous projects. When the opportunity to apply for a violence prevention center emerged, the first step was to convene a meeting of representatives from local organizations to discuss the proposal. The investigators also reached out to new organizational partners with expertise in youth development and violence prevention. In these initial meetings, the group identified the geographic area of focus and the intervention strategies. These early meetings helped build a foundation of trust among existing and new partners, and provided opportunities to identify mutual goals and outcomes even before funding was received.

##### Transparency

After the grant was awarded, the initial planning group became the nucleus of the Center’s Steering Committee. The Steering Committee advised the MI-YVPC investigators, disseminated data and materials from the Center and served as a venue for organizations to share information about their own programs and events. Regular interaction with the Steering Committee on the progress of the interventions and evaluation findings helped to ensure the transparency of the Center’s operations. To further promote transparency, the Center established written agreements with participating organizations that clearly delineated their roles and responsibilities and what compensation they would receive.

##### Communication

The Center faced a communication challenge in reaching out to community members who were not directly involved in its activities. While Flint has many active neighborhood groups, most residents are not formally connected to organizations. The MI-YVPC took advantage of its data collection efforts to communicate about the Center and potentially engage residents in its interventions. Each summer, local research assistants from the Center walked through the intervention area to rate property parcels on a number of parameters (Reischl et al., 2014). These “parcel assessors” wore MI-YVPC t-shirts and carried brochures about the Center. In their frequent encounters with residents, they explained the program and provided literature. In the course of these conversations, they also learned about residents’ concerns and priorities and passed this information along to the Steering Committee.

To further extend the reach of youth engagement beyond structured interventions, Michigan YVPC supported outreach events to involve youth in activities that promote positive development, while raising the awareness and visibility of the Center. The Center hosted a Safe and Healthy Futures youth festival that included performances by local youth groups, and a Safe and Healthy Futures contest for fifth and sixth graders who created art work and essays about their goals and aspirations. The winning entries were displayed publically at different venues around the city. In addition the Center staff attended numerous health fairs and other community events and maintained an active presence on its web-site and social media. These varied approaches provided opportunities for the Center to communicate its mission to the broader community.

##### Commitment

Individuals and organizations located in communities with limited resources are often over-extended. It is difficult for them to commit to participation unless they see a clear benefit. In such settings there is competition for resources, and a new initiative may be viewed as a rival for attention and funding. The MI-YVPC worked closely with community organizations that had established track records, thereby demonstrating a commitment to building local capacity and sustainability. The Center employed staff from the local community whenever possible and benefited from their experiences and perspectives.

The Center built on existing community assets by supporting several local organizations that took the lead on program implementation: The Genesee County Land Bank Clean and Green program engaged neighborhood groups to maintain and improve vacant properties. The Boys and Girls Club of Greater Flint provided a mentoring program developed by the Boys and Girls Club of America. A local church implemented the Youth Empowerment Solutions program that prepared youth to design and carry out community improvement projects (Zimmerman, Stewart, Morrel-Samuels, Franzen, & Reischl, 2011). Faculty and staff from the Michigan State University School of Criminal Justice worked on community mobilization with neighborhood groups, providing crime prevention information and helping these organizations obtain funding to clean up and maintain vacant properties. The Fathers and Sons program employed local facilitators to implement its family-focused curriculum (Caldwell, Rafferty, Reischl, De Loney, & Brooks, 2010). Project Sync screened and counseled adolescents who visited the emergency department of a local hospital, a strategy that reached many who might never have participated in a structured program. Each of these collaborations enhanced capacity, provided local investment, and promoted sustainability.

To demonstrate commitment, the MI-YVPC also served as a resource for community organizations’ own programs and activities and was a visible presence at community events and other coalitions. For example, data collected by the MI-YVPC was shared widely within the community and supported the Flint Master Plan and the health department’s strategic planning process. The Center participated in the University Avenue Coalition (UACC) that works to address blight in an overlapping area and participated in proposals to secure funding for the UACC. These activities increased the Center’s reach, added value to locally-driven efforts and demonstrated the Center’s commitment to the broader community.

## Discussion

Social ecological and social disorganization theories suggest that externally driven violence prevention efforts that do not include community engagement are unlikely to influence the social norms and conditions that affect youth violence (Gorman-Smith et al., 2004; Kawachi, & Kennedy, 1999; Peterson, & Newman, 2000; Sampson et al., 2002; Taylor et al., 1984). Well planned and carefully implemented community engagement methods are necessary to create positive contexts and resources for positive youth development and violence prevention.

Comprehensive strategies to prevent violence bring together disparate partners, including public health, law enforcement, education, and community-based organizations and residents. Although these diverse partnerships provide access to multiple resources and varied expertise, they may also be hampered by misunderstandings and mistrust. Establishing trust is a key component of successful youth violence prevention collaborations (Griffith et al., 2008; Kim-Ju et al., 2008). This requires devoting sufficient time and attention to relationship building and establishing key connections with local organizations. Each of the Centers described here used different approaches to establish trusting relationships. Investigators from the NC-YVPC had to build relationships from the ground up in an area of the state where they had not worked before. The NC-YVPC’s investigators found that using trusted local intermediaries helped them to establish relationships with community leaders and residents. In Colorado, YVPC leaders chose the structured “Communities That Care” model to guide their community engagement efforts. This model provided a roadmap for working in a new community with a diverse population and changing dynamics. The MI-YVPC had a number of well-established relationships in Flint, but was challenged to bring new partners to the table to enrich its community ties. In a concentrated urban area, such as Flint, local organizations may be well-connected, but may also have turf issues or other barriers to collaboration that require continued attention to trust-building.

Transparency is described as a critical component of successful partnerships (Green et al., 2001). Clarity concerning the roles of the various partners, plans for the future, resources, and expectations may forestall potential misunderstandings. Being transparent about the known duration of a specific project or initiative will help prevent disappointment, as will a commitment to share existing resources and promote sustainability. Each of the Centers convened advisory groups comprising a wide range of local representatives that helped to establish the goals and methods of the violence prevention initiatives. The NC-YVPC convened a Youth Violence Prevention Council that worked on developing a mission, goals and partnership processes. The Communities That Care model, used by the CO-YVPC, provided a blueprint for community engagement in setting goals. In Flint, the MI-YVPC solicited community partners’ input concerning the goals articulated in the proposal to the funder. In many instances, formal agreements were established with schools, agencies and community organizations outlining the responsibilities of all parties. These practices contributed to ensuring clarity and shared understanding among all partners.

Frequent and consistent communication among partners is necessary to build and sustain collaborative work (Plowfield et al., 2005). While youth’s health and safety is a shared community priority, differences in perspectives, cultures and disciplines may interfere with clear communications and understandings. The forms of communication should be in keeping with local social norms and values. Email and conference calls may work in certain circumstances, but many community members prefer face-to-face meetings, particularly early in the relationship building process. In rural North Carolina, an informal introduction at a restaurant was preferred to a scheduled meeting in the office. The same may not be true in a community where the boundaries between professional and personal life are more distinct. The CO-YVPC found that consistently attending, supporting, and contributing to a variety of community meetings and events showed an interest in other relevant community issues and created a mechanism for neighborhood leaders to engage in dialogue about youth violence prevention as it related to their own initiatives. Concerted communication efforts with the Hispanic community resulted in their eventual partnership on one of the CO-YVPC interventions. Proactive communication can help to establish the credibility and visibility of partnerships. The MI-YVPC, for example, made data and information gathered by the Center widely available to inform other local initiatives such as the master planning process, thus leading to broader community support.

Communities are rightly concerned about commitment (Green et al., 2001; Plowfield et al., 2005). Too often organizations get involved in initiatives, only to see the effort abandoned if funding is not forthcoming. Each of the Centers has employed experienced staff from the community and contracted with local organizations to implement interventions. Local residents have helped to collect data and lead programs. These practices build capacity, enhance the sustainability of violence prevention work, and contribute to the local economy. They exemplify the principle of building upon existing community assets and structures articulated by Green et al. (2001). The NC-YVPC demonstrated its commitment by adapting the selected evidence-based interventions to respond to community values and concerns.

Just as evidenced-based programs may need to be adapted in order to meet the needs of specific populations, methods of community engagement must also be tailored to the contexts in which they will occur. Each of these case examples includes descriptions of a fifth principle that appears to be critical to partnership success: flexibility. Successful community engagement requires flexibility to take advantage of emerging opportunities, to adopt new strategies when needed, and to embrace the guidance of local collaborators. On the advice of local partners, the NC-YVPC tailored aspects of their interventions to better align with the cultural context of the community. When faced with difficulties connecting with the Hispanic community, the CO-YVPC took advantage of invitations to promote its work at community events and to recruit local staff members. The MI-YVPC decided to use its data collection efforts to engage residents directly by training local research assistants to provide information and listen to residents’ concerns and perspectives. While youth engagement is critical to any violence prevention efforts, reaching adolescents in particular can present many obstacles. Structured youth violence interventions or youth committees may fail to engage the youth who are most at risk. Many youth may never join a formal program but may still be affected by environmental interventions, community events or summer employment opportunities. Flexibly engaging youth in natural settings can be an important supplement to structured violence prevention interventions. The MI-YVPC created informal opportunities for youth engagement in community events and contests with violence prevention themes.

These case examples are drawn from three centers that serve different populations within varied contexts. While they illustrate some commonalities and differences across sites, the conclusions drawn from the examples may not be generalizable to all violence prevention collaborations. Future research could include a formal case study design involving a larger sample of partnerships to further advance our understanding of the dynamics of community engagement in violence prevention. Nevertheless, these examples provide information that may be useful to emerging youth violence coalitions.

## Conclusion

The varying geographies, histories, and racial and ethnic compositions of these three YVPCs offer unique challenges and opportunities for collaborative partnerships. However, as documented in the literature, and illustrated by these case examples, there are certain principles of community engagement that appear to be common across populations and geography. They may be summarized under the broad categories of trust, transparency, communication, and commitment, all of which are interconnected. They also suggest another aspect of successful community engagement, flexibility. The case examples presented in this article illustrate some of the benefits and challenges of applying principles of community engagement to comprehensive youth violence prevention initiatives. First and foremost, trust and transparency are pre-requisites for gaining the cooperation and participation of local communities in violence prevention work. Trusting relationships among academic and community organizations provide avenues for raising awareness, recruiting participants, and incorporating local knowledge and expertise. Open communication undergirds opportunities for bi-directional capacity-building among partners: academics bring familiarity with data and evaluation methods, while practitioners and residents contribute an intimate understanding of community needs, values and contextual conditions. Investments in local capacity lay the foundation for sustainability of programs and environmental interventions and also demonstrate commitment to the community over the long term. Finally, community engagement methods must be flexible so as to be responsive to changing conditions, new learning and serendipitous opportunities.
